# Functional Connectivity of the Dorsal and Ventral Attention Network and Its Role in Attentional Disengagement

**DOI:** 10.1002/brb3.70868

**Published:** 2025-09-28

**Authors:** Sélim Yahia Coll, Emilie Marti, Naz Doganci, Radek Ptak

**Affiliations:** ^1^ Laboratory of Cognitive Neurorehabilitation, Faculty of Medicine University of Geneva Geneva Switzerland; ^2^ Division of Neurorehabilitation, Department of Clinical Neurosciences University Hospitals of Geneva Geneva Switzerland

**Keywords:** attention networks, disengagement, resting‐state functional connectivity, spatial attention

## Abstract

**Background:**

The interplay between the ventral attention network (VAN) and dorsal attention network (DAN) is crucial for attentional control, particularly in disengagement processes. While task‐based fMRI studies have extensively characterized their roles, less is known about whether intrinsic connectivity patterns during rest within these networks predict individual differences in attentional disengagement. This study investigates the relationship between resting‐state functional VAN‐DAN connectivity and disengagement efficiency.

**Methods:**

An initial sample of 85 healthy participants completed a spatial cueing task, assessing attentional disengagement through reaction time differences between valid and invalid cue trials. Resting‐state fMRI data were collected and analyzed using seed‐based connectivity methods. Functional connectivity between key VAN and DAN regions—frontal eye fields (FEF), intraparietal sulcus (IPS), inferior frontal gyrus (IFG), and supramarginal gyrus (SMG)—was examined in relation to a disengagement index, representing cue validity effects.

**Results:**

Participants exhibited slower responses to invalidly cued targets, with a greater disengagement cost in the left visual field. Functional connectivity analyses revealed that VAN regions, particularly the right IFG and SMG, showed stronger associations with attentional disengagement than DAN regions. Increased FC with occipito‐temporal areas correlated with heightened validity effects in the left hemifield, while greater connectivity with medial parietal and cingulate regions was linked to reduced disengagement asymmetry. Interhemispheric connectivity also played a modulatory role in attentional control.

**Conclusion:**

These findings underscore the role of VAN over DAN in attentional disengagement, emphasizing the right IFG and SMG in reorienting attention. Greater connectivity with occipito‐temporal regions may hinder disengagement, while enhanced functional connectivity with medial cortical areas facilitates adaptive shifts in attention. This study highlights the importance of intrinsic VAN‐DAN interactions in shaping attentional control and provides insights into the neural mechanisms underlying disengagement efficiency.

## Graphical abstract

Resting‐state connectivity within the ventral and dorsal attention network, particularly in the right hemisphere, predicts individual differences in attentional disengagement efficiency, highlighting the role of intrinsic brain dynamics in shaping attentional control.

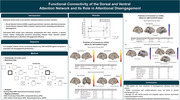

## 1 Introduction

Posner's attention network model delineates three core systems underlying attentional control: alerting, orienting, and executive control (Posner and Petersen 1990). The orienting network, in particular, comprises three critical components (Posner 1984): (1) engagement, wherein attention is allocated to a novel stimulus; (2) disengagement, involving the release of attentional focus from a previously attended location; and (3) shifting, which facilitates the redirection of attention toward a new target. These processes have been extensively characterized using the spatial cueing paradigm (Posner 1980), in which participants respond to peripherally presented targets following cues that may either validly or invalidly predict target location. This paradigm has been instrumental in dissecting the functional architecture of attentional orienting and its underlying neural mechanisms.

A dynamic interplay between the ventral attention network (VAN) and dorsal attention network (DAN) has been implicated in attentional control (Corbetta and Shulman 2002). The VAN, comprising the supramarginal gyrus (SMG), inferior frontal gyrus (IFG), and insular cortex (Corbetta and Shulman 2002) is primarily associated with stimulus‐driven, bottom‐up attentional shifts (Vossel et al. 2014). The DAN encompasses the frontal eye fields (FEF) and intraparietal sulcus (IPS; Corbetta and Shulman 2002) and is integral to goal‐directed, top‐down attentional modulation (Vossel et al. 2014). While the DAN is believed to exert control similarly for stimuli appearing in left or right space, damage to brain regions overlapping with the VAN leads to strongly lateralized impairments of spatial attention, primarily interpreted as a deficit of attentional disengagement and reorienting (Carter et al. 2017; Friedrich et al. 1998; Losier and Klein 2001; Morrow and Ratcliff 1988; Posner et al. 1984; Ptak and Bourgeois 2024). Advanced lesion‐symptom mapping in spatial neglect has implicated ventral regions, notably the right temporoparietal junction (TPJ) and right insular cortex, in attentional reorienting (Coll et al. 2024; Ptak and Bourgeois 2024; Ptak and Pedrazzini 2021). The prevailing model suggests that VAN‐mediated detection of salient, competing stimuli (Corbetta and Shulman 2002; Shomstein et al. 2010; Vossel et al. 2014) triggers an interruptive signal to the DAN, thereby facilitating the reallocation of attentional resources (Shomstein et al. 2010; Vossel et al. 2014).

To date, most investigations of VAN‐DAN interactions have utilized task‐based fMRI, wherein attentional processes are examined within active task conditions (Corbetta and Shulman 2002; Fox et al. 2006; Posner and Petersen 1990). While such approaches enable direct mapping of behavior to neural activity, they do not address whether the functional architecture of these networks is exclusively task‐dependent or also reflected in intrinsic connectivity patterns during rest. Resting‐state fMRI (rs‐fMRI) studies have demonstrated that the VAN and DAN exhibit distinct functional connectivity (FC) profiles even in the absence of task demands (Fox et al. 2006), prompting investigations into whether resting‐state functional coupling of these networks can predict attentional performance. For example, Wen et al. (2012) demonstrated that Granger causal influences between the VAN and DAN predict attentional reorienting efficiency, and Machner et al. (2022) identified associations between DAN connectivity (e.g., FEF‐IPS interactions) and behavioral outcomes in spatial attention tasks. Additional studies have linked frontoparietal network connectivity to the alerting and executive control components of attention (Markett et al. 2014) and have implicated right TPJ connectivity in belief updating during attentional shifts (Käsbauer et al. 2020).

Despite these findings, few studies have specifically investigated rs‐fMRI connectivity within VAN and DAN regions in relation to attentional disengagement. Becker et al. (2024) reported increased VAN connectivity in children exhibiting cognitive disengagement symptoms, whereas Lissek and Tegenthoff (2021) explored DAN contributions to disengagement in extinction renewal paradigms. The only study directly investigating VAN‐DAN interactions in spatial cueing tasks, to our knowledge, was conducted by Russman Block et al. (2017), who found that stronger VAN‐DAN connectivity correlated with successful disengagement in healthy controls but was disrupted in PTSD populations.

The present study extends this line of research by investigating the role of intrinsic VAN‐DAN connectivity in attentional disengagement within a neurotypical population. Using rs‐fMRI, we aim to determine whether FC patterns within these networks predict individual differences in disengagement efficiency, as measured by performance in a spatial cueing paradigm.

## 2 Materials and Methods

### 2.1 Participants

An initial sample of 85 healthy right‐handed participants took part in our study. They all reported no current or previous neurological or psychiatric disorders. Out of them, 19 individuals refused to participate in the fMRI part of the protocol and were thus discarded. Seven additional participants were excluded due to issues during testing or MRI scanning, such as excessive head movement or missing behavioral or functional data. Therefore, 59 participants (27 females; mean age = 49.20 ± 19.50) were included in the final analyses. Informed consent was obtained from each participant, and the study was approved by the Ethical Commission of the Canton of Geneva, in accordance with the Declaration of Helsinki.

### 2.2 Study Procedure and Task

**FIGURE 1:**
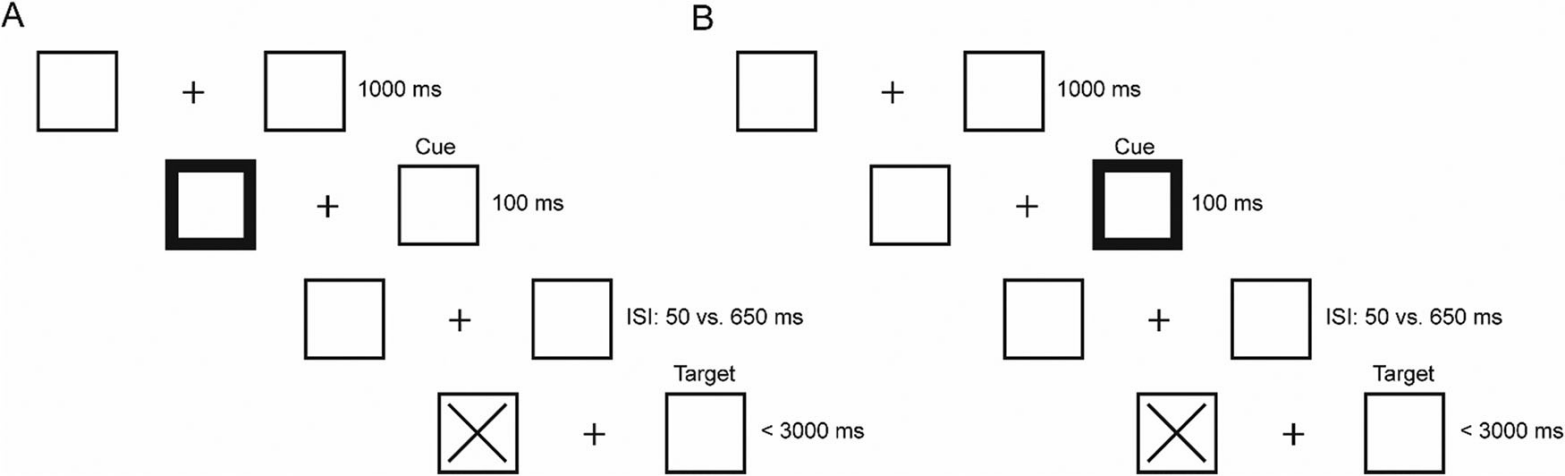
Sequence and timing of events of the spatial attention task. (A) Example of a valid trial. (B) Example of an invalid trial. ISI = interstimulus interval.

The study comprised two sessions: a behavioral session to examine spatial attention using a cueing task, and an MRI session. The task was programmed using E‐Prime 2.0 software (Psychology Software Tools, Pittsburgh, PA) and was an adaptation of the original Posner cueing paradigm (Posner 1980; See Figure 1). All stimuli were displayed on a 13‐inch laptop (resolution: 1280 × 800 pixels) with a neutral gray background. Two peripheral squares (size: 150 × 150 pixels) were continuously presented, positioned 25% from the left or right edges of the screen. A cue was presented as a brief thickening (100 ms) of the edge of one of the squares, followed by the target (a large “X”) after either a 50 or 650 ms delay, corresponding to stimulus‐onset asynchronies (SOAs) of 150 or 750 ms, respectively. Three‐quarters of the trials featured short SOAs, while one‐quarter included long SOAs, which were intended to prevent anticipatory responses and were excluded from further analysis.

Participants were seated approximately 50 cm from the laptop and instructed to maintain their gaze on a small fixation cross presented at the center of the screen. They were asked to respond as quickly as possible by pressing a button on a Cedrus response box (Cedrus, San Pedro, CA) upon target appearance. To ensure proper fixation, participants completed 20 practice trials before the experiment commenced.

The experiment consisted of three blocks, each containing 64 trials. The trials included an orthogonal combination of cue direction (left or right) and target side (left or right). Importantly, the cue was not predictive of the target's location.

MRI scans were performed on a 3‐T Prisma Fit scanner with a 64‐channel array coil (Siemens Medical Solutions, Erlangen, Germany) at the Center for Biomedical Imaging, University Hospitals of Geneva. Participants were positioned inside the scanner with their eyes open while both structural and functional images of the entire brain were acquired. Structural images were obtained using a high‐resolution T1‐weighted MPRAGE sequence with the following parameters: repetition time (TR) = 2300 ms, echo time (TE) = 1.96 ms, number of slices = 176, voxel size = 1.0 mm isotropic, and flip angle = 9°. Resting‐state functional images were acquired using a fast echo planar imaging (EPI) sequence with TR = 720 ms, TE = 30 ms, number of volumes = 440, voxel size = 2.5 mm isotropic, and flip angle = 50°.

#### 2.2.1 Behavioral Analysis

The behavioral analysis focused on reaction times (RTs) to targets shown at shorter SOAs (150 ms), as these are associated with larger cue validity effects (Müller and Findlay 1988). Trials with RTs exceeding two standard deviations from each participant's mean were excluded. A repeated‐measures analysis of variance was conducted on the median RTs, with the factors cue validity (valid vs. invalid) and target side (left vs. right). To control for potential age effects, participant's age was included as a covariate in an additional repeated‐measures analysis of covariance (ANCOVA).

#### 2.2.2 Resting‐State fMRI Analysis

Functional data were analyzed using a preprocessing pipeline implemented in the CONN toolbox (Whitfield‐Gabrieli and Nieto‐Castanon 2012). Functional scans were first aligned by coregistering all images to a reference scan and resampling to correct for motion artifacts and magnetic susceptibility distortions. Slice timing correction was performed via sinc temporal interpolation to align the BOLD timeseries to a common mid‐acquisition time. Artifact detection tools (ART) were used to identify outlier scans (Power et al. 2014), and a representative BOLD image for each subject was created by averaging scans while excluding outliers.

Spatial normalization to the MNI standard space was carried out using SPM12's unified segmentation and normalization approach (http://www.fil.ion.ucl.ac.uk/spm), followed by Gaussian smoothing with an 8 mm FWHM kernel. Denoising involved regressing out confounding factors, including white matter and cerebrospinal fluid (CSF) timeseries, motion parameters, outlier scans, session‐specific effects, and linear trends within each run. BOLD timeseries were bandpass filtered within a frequency range of 0.008–0.09 Hz. Noise components from white matter and CSF, estimated using CompCor (Behzadi et al. 2007), helped refine the degrees of freedom in the cleaned BOLD signal.

FC strength was quantified using Fisher‐transformed bivariate correlation coefficients, calculated with a weighted general linear model (GLM; Nieto‐Castanon 2020). For group‐level analysis, a GLM approach was applied to evaluate voxel‐level hypotheses using multivariate parametric tests. Cluster‐level inferences were drawn using Gaussian Random Field theory, with results thresholded at voxel‐level significance of *p* < 0.001 and a family‐wise error‐corrected cluster size threshold of *p*‐FDR < 0.05.

The relationship between resting‐state functional connectivity (FC) and behavior is generally established on statistical correlation and therefore requires simple measures of performance. To obtain a simple measure of attention disengagement we computed a disengagement index (DI) with the following formula: DI = (RT_left‐invalid −_ RT_left‐valid_)/(RT_right‐invalid −_ RT_right‐valid_). Positive values indicate stronger effects of cue validity in the left visual field than the right visual field, while the inverse is true for negative values.

The relationship between FC and DI was examined using seed‐based analyses. Given the well‐established dominance of the right cerebral hemisphere in attention, we selected four seed regions located in the right hemisphere's VAN and DAN, which play a pivotal role in attentional disengagement (Corbetta and Shulman 2002). Two of these seed regions (IPS; FEF) were part of the DAN, while the other two (IFG; SMG) were associated with the VAN. The definitions of IPS and FEF were based on the network atlas included in the CONN toolbox. The definitions of IFG and SMG were based on the Harvard–Oxford cortical atlas, combining the opercular and triangular parts of the IFG and the anterior and posterior portions of the SMG, respectively.

FC between these seed regions and the entire brain, including both cortical and subcortical areas, was investigated. To account for potential age‐related effects on the FC‐behavior relationship, age was included as a covariate in all analyses.

## 3 Results

### 3.1 Behavioral Results

**FIGURE 2:**
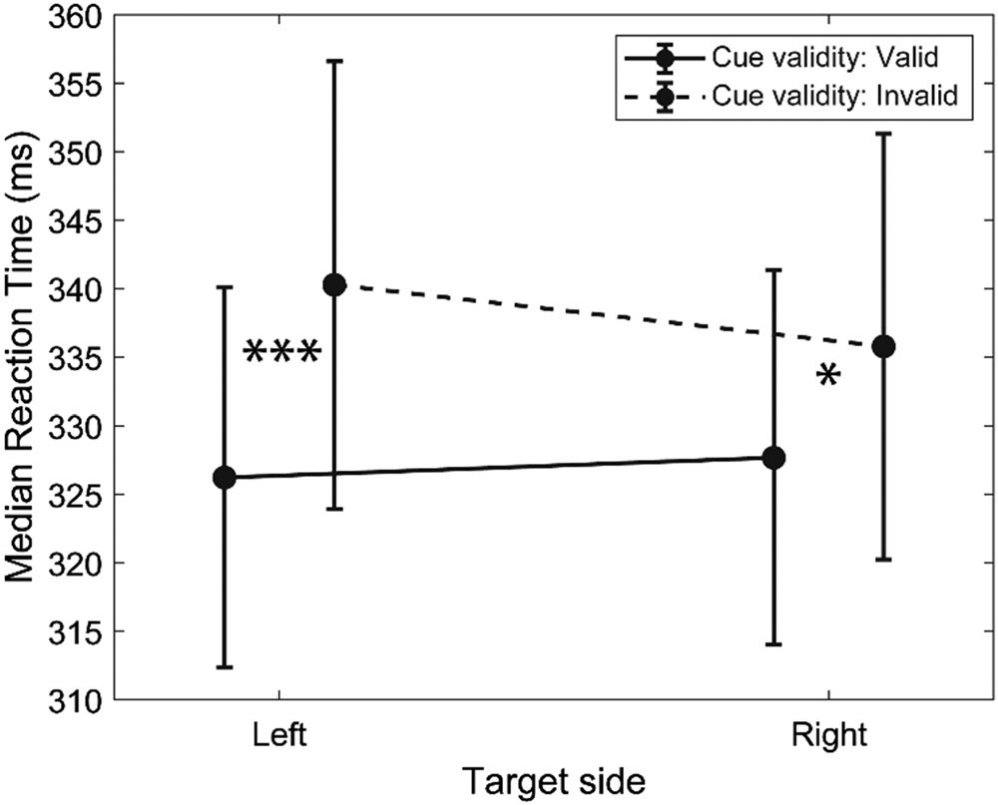
Plot of the two‐way interaction between target side (left vs. right) and cue validity (valid vs. invalid) without controlling for the age of participants. Vertical bars denote 95% confidence intervals. *** = difference between valid and invalid cues with *p* < 0.001, * = difference between valid and invalid cues with *p* < 0.05.

Figure 2 shows the behavioral results. The repeated‐measures ANOVA yielded no effect of Target side (*F*_1,58_ = 0.50, *p* = 0.481, *η^2^_p_* = 0.009), but a significant effect of cue validity (*F*_1,58_ = 14.76, *p* < 0.001, *η^2^_p_* = 0.203), indicating that participants were slower in the invalid than the valid condition. The interaction between target side and cue validity was also significant (*F*_1,58_ = 4.09, *p* = 0.048, *η^2^_p_* = 0.066). False discovery rate‐corrected simple effects (Benjamini and Yekutieli 2005) revealed that although participants exhibited significant validity effects for both target sides (left: *t*_58_ = 4.54, *p* < 0.001; right: *t*_58_ = 2.40, *p* = 0.020), the validity effect for left targets was greater than for right targets (*t*_58_ = 2.02, *p* = 0.048).

Considering the large age range of our sample, we decided to repeat the same analysis by including age as a covariate. The ANCOVA showed indeed that age had a significant effect, justifying its inclusion as a continuous predictor (*F*_1,57_ = 37.04, *p* < 0.001, *η^2^_p_* = 0.394). However, after controlling for age the cue validity (*F*_1,57_ = 0.86, *p* = 0.359, *η^2^_p_* = 0.015), target side (*F*_1,57_ = 0.04, *p* = 0.835, *η^2^_p_* = 0.001), and interaction effects (*F*_1,57_ = 0.63, *p* = 0.430, *η^2^_p_* = 0.011) failed to reach significance.

### 3.2 Resting‐State fMRI Results

Before presenting the fMRI results, it is important to clarify the interpretation of FC‐behavior correlations. A positive correlation between FC and DI indicates a larger validity effect for stimuli presented in the left hemifield, meaning an increased cost of invalid cues in the right hemifield. Conversely, a negative correlation suggests a larger validity effect in the right hemifield, implying a higher cost of invalid cues in the left hemifield.

#### 3.2.1 Predictors of Increased Cue Validity Effects for Left Hemifield Targets

**FIGURE 3:**
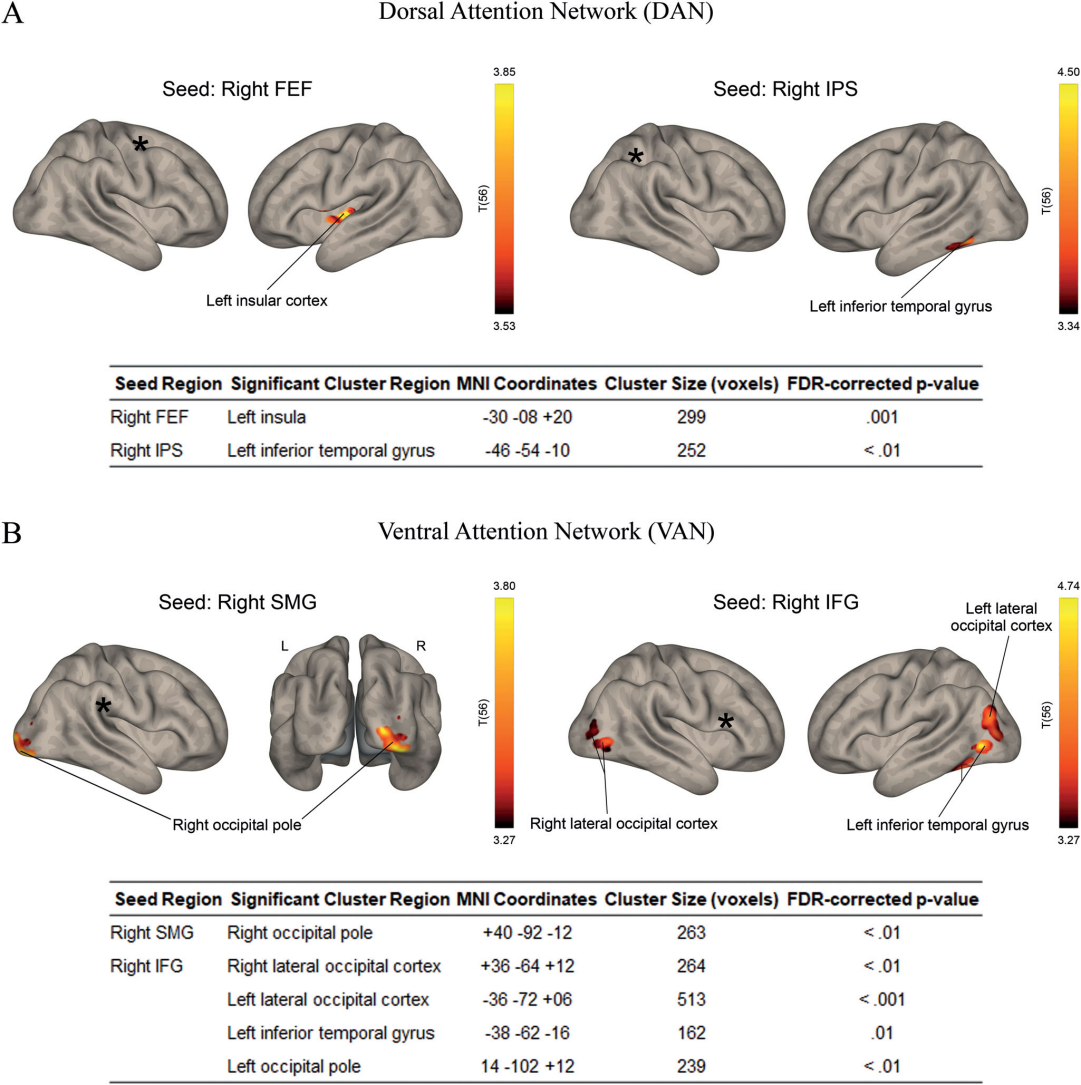
Resting‐state fMRI results showing the significant (cluster threshold: *p* < 0.05 cluster‐size *p*‐FDR corrected) predictors of increased cue validity effects for left hemifield targets. (A) Seeds in the dorsal attention network (DAN): right frontal eye field (FEF) and right intraparietal sulcus (IPS). (B) Seeds in the ventral attention network (VAN): right supramarginal gyrus (SMG) and right inferior frontal gyrus (IFG).

Analysis of the DAN identified two significant functional connections predicting increased cue validity effects for left hemifield targets (Figure 3A). One connection was between the right FEF and left insula (cluster size: 299 voxels; MNI coordinates: −30, −08, +20; FDR‐corrected *p* = 0.001), while the other was between the right IPS and left inferior temporal gyrus (cluster size: 252 voxels; MNI coordinates: −46, −54, −10; FDR‐corrected *p* < 0.01) as positive predictor of the DI.

In contrast, the VAN showed a more complex pattern (Figure 3B). A positive FC‐DI correlation was observed between the right SMG and the right occipital pole (cluster size: 263 voxels; MNI coordinates: +40, −92, −12; FDR‐corrected *p* < 0.01). More importantly, the right IFG exhibited widespread positive correlations with bilateral posterior brain areas, including the right (cluster size: 264 voxels; MNI coordinates: +36, −64, +12; FDR‐corrected *p* < 0.01) and left lateral occipital cortex (cluster size: 513 voxels; MNI coordinates: −36, −72, +06; FDR‐corrected *p* < 0.001), the left inferior temporal gyrus (cluster size: 162 voxels; MNI coordinates: −38, −62, −16; FDR‐corrected *p* = 0.01), and the left occipital pole (cluster size: 239 voxels; MNI coordinates: −14, −102, +12; FDR‐corrected *p* < 0.01). These connectivity patterns were associated with an increased cost of invalid cues for targets in the left hemifield.

#### 3.2.2 Predictors of Increased Cue Validity Effects for Right Hemifield Targets

**FIGURE 4:**
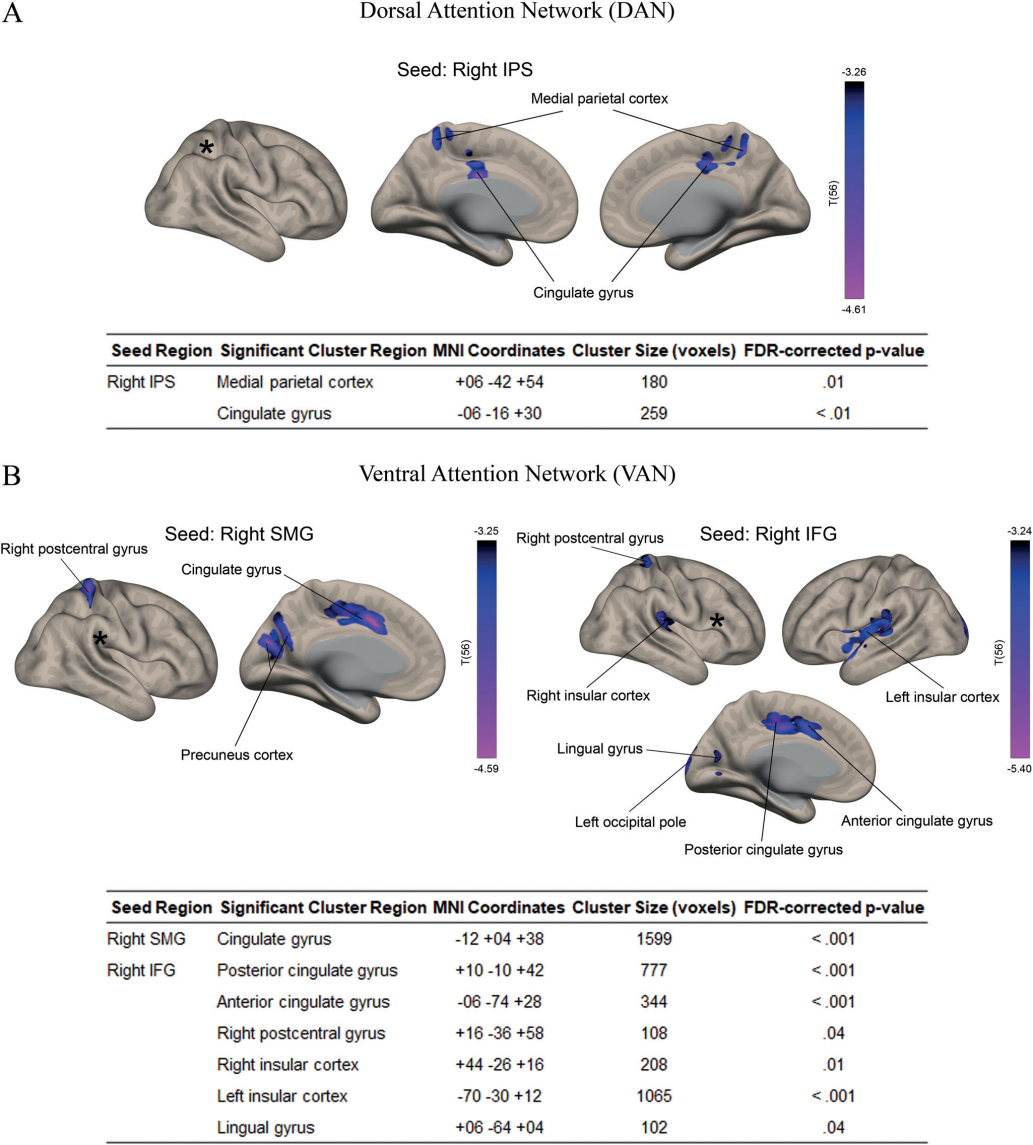
Resting‐state fMRI results showing the significant (cluster threshold: *p* < 0.05 cluster‐size *p*‐FDR corrected) predictors of increased cue validity effects for right hemifield targets. (A) Seeds in the dorsal attention network (DAN): right frontal eye field (FEF) and right intraparietal sulcus (IPS). (B) Seeds in the ventral attention network (VAN): right supramarginal gyrus (SMG) and right inferior frontal gyrus (IFG).

Within the DAN (Figure 4A), the right IPS seed revealed two bilateral brain regions whose connectivity correlated negatively with DI, predicting an increased cost of invalid cues for right hemifield targets: the medial parietal cortex (cluster size: 180 voxels; MNI coordinates: +06, −42, +54; FDR‐corrected *p* = 0.01) and the cingulate gyrus (cluster size: 259 voxels; MNI coordinates: −06, −16, +30; FDR‐corrected *p* < 0.01).

Within the VAN (Figure 4B), a cluster in the cingulate gyrus also exhibited connectivity with the SMG (cluster size: 1599 voxels; MNI coordinates: −12, +04, +38; FDR‐corrected *p* < 0.001) and two clusters with the IFG (cluster size: 777 voxels; MNI coordinates: +10, −10, +42; FDR‐corrected *p* < 0.001; cluster size: 344 voxels; MNI coordinates: −06, −74, +28; FDR‐corrected *p* < 0.001). In addition, the IFG showed significant FC‐behavior correlations with several other clusters, including the right postcentral gyrus (cluster size: 108 voxels; MNI coordinates: +16, −36, +58; FDR‐corrected *p* = 0.04), the right (cluster size: 208 voxels; MNI coordinates: +44, −26, +16; FDR‐corrected *p* = 0.01) and left (cluster size: 1065 voxels; MNI coordinates: −70, −30, +12; FDR‐corrected *p* < 0.001) insula, and the lingual gyrus (cluster size: 102 voxels; MNI coordinates: +06, −64, +04; FDR‐corrected *p* = 0.04).

## 4 Discussion

The current study examined Rs‐fMRI connectivity within the DAN and VAN and their predictive relationship with disengagement effects, as measured by a spatial cueing paradigm. Both behavioral and Rs‐fMRI analyses provided key insights into disengagement processes. Specifically, participants exhibited slower responses when an invalid cue indicated a target's location in the left compared to the right visual field. This larger cost for left hemifield targets aligns with the right hemisphere's dominance in attention, particularly in alerting and orienting mechanisms (Fan et al. 2005; Siman‐Tov et al. 2007; Singh‐Curry and Husain 2009). Although the cue validity effect did not reach statistical significance after controlling for age—consistent with findings in healthy populations (e.g., Coll et al. 2024)—this does not undermine the rationale of our approach. Our goal was not to demonstrate a group‐level effect, but to examine how individual differences in disengagement efficiency relate to intrinsic FC. In fact, testing this hypothesis requires sufficient variability across participants, including individuals with both high‐ and low‐disengagement costs. It should also be noted that while participants were screened for neurological and psychiatric conditions through self‐report, no standardized clinical assessments (e.g., ADHD or depression scales) were administered. Although this limits the ability to account for potential subclinical traits, such measures are more relevant to questions of distractibility or sustained attention, which fall outside the scope of the present study.

In this regard, the DI was associated with distinct connectivity patterns involving seed regions in both the DAN and VAN. Connectivity analyses revealed three main findings: (1) VAN seeds identified more brain regions whose connectivity predicted disengagement effects than DAN seeds; (2) increased DI was linked to heightened FC in occipito‐temporal areas, whereas decreased DI corresponded to greater FC in the insula, postcentral gyrus, and medial cortices (cingulate gyrus and precuneus); and (3) decreased DI was associated with increased interhemispheric connectivity.

As regards the first result, our findings support the greater involvement of the VAN over the DAN in attentional disengagement, consistent with previous research. Functional imaging studies using variants of the Posner paradigm indicate widespread activation of dorsal and ventral fronto‐parietal networks, yet the right inferior parietal lobe and temporo‐occipital junction (TPJ) appear particularly sensitive to invalid cues (Corbetta et al. 2000; Fan et al. 2005; Indovina and Macaluso 2007). Corbetta et al. (2000) reported the strongest validity‐related responses in the right TPJ, while Kincade et al. (2005) demonstrated that the SMG and IFG are strongly activated when attention is reoriented following an invalid cue. The right TPJ also exhibits heightened responses to distractors that share a target‐defining feature, such as color (Serences et al. 2005), reinforcing its role in orienting toward behaviorally salient stimuli. Corbetta et al. (2008) therefore proposed that the TPJ is crucial for reorienting to unexpected and particularly salient environmental stimuli. Lesion studies further support this role, as spatial neglect—a disorder characterized by impaired attentional reorienting—has been linked to damage in the inferior parietal lobe and TPJ (Golay et al. 2008; Losier and Klein 2001; Pedrazzini and Ptak 2020; Ptak and Bourgeois 2024). Such lesions are associated with deficits in disengaging attention from ipsilateral cues following right‐hemispheric injury (Bourgeois et al. 2022; Coll et al. 2024; Pedrazzini and Ptak 2019; Ptak and Pedrazzini 2021), precisely in the regions showing strong activation in response to invalid spatial cues.

Although less prominent than the TPJ, the IFG also plays a significant role in spatial orienting. This region consistently shows activation in spatial cueing tasks, particularly when evaluative and controlled processes are required (Cieslik et al. 2015; Han and Marois 2014). Moreover, damage to the IFG has been linked to spatial neglect and increased validity effects for contralateral visual targets (Coll et al. 2024; Husain and Kennard 1996; Ptak and Schnider 2010). These findings provide converging evidence that the two VAN seed regions contribute substantially to attentional disengagement. Given this, it is unsurprising that FC involving VAN seeds was particularly associated with performance in the spatial orienting task.

Perhaps the most novel and intriguing finding of our study is that several right‐hemisphere seeds exhibited connectivity with medial brain regions that correlated with a decreased DI. Specifically, the SMG and IFG connected to the cingulate gyrus and precuneus, while the IPS showed FC with the cingulate and medial parietal cortex. Previous functional imaging studies have demonstrated that the posterior cingulate cortex exhibits cue‐related activity, particularly when the cue accurately predicts the target's location (Hopfinger et al. 2000; Mesulam et al. 2001; Small et al. 2003). In contrast, anterior cingulate activity is more closely associated with target detection. As part of the limbic system, the cingulate cortex receives anatomical inputs from the superior and lateral parietal cortex as well as anterior limbic structures, such as the orbitofrontal cortex (Rolls 2019). Its primary role in attention has been interpreted as integrating motivational value and orienting signals to mediate expectancy and the motivational salience of significant events (Small et al. 2003). While resting‐state connectivity alone does not allow for definitive conclusions about the precise role of a given brain region, the observed interactions between the DAN, VAN, and cingulate cortex align with this interpretation.

A second major finding of our study is that an increased validity effect for left hemifield targets was associated with greater FC in occipito‐temporal brain areas, whereas a decreased DI corresponded to stronger FC in the cingulate gyrus, precuneus, and insula. Previous research has shown that the striate and extrastriate cortices exhibit increased activity during directed attention, even in the absence of visual stimulation (Kastner et al. 1999; Watanabe et al. 2011). Since activation in these regions is largely automatic, it likely reflects a bottom‐up, exogenous mode of processing. In our study, their connectivity with the VAN was linked to greater spatial asymmetry in attention deployment, potentially relating to the right‐hemispheric lateralization of the VAN and its dominance in spatial attention. In contrast, increased FC with regions outside the occipito‐temporal cortex—specifically the precuneus, cingulate cortex, and insula—was associated with reduced left spatial bias, suggesting that these areas play a modulatory role in attentional reorienting.

The involvement of these regions is also central to the third major finding of our study: a decreased validity effect for left hemifield targets was associated with increased interhemispheric connectivity. This result particularly supports the idea that coupling between the right‐hemisphere VAN and the left‐hemisphere precuneus, cingulate cortex, and insula plays a modulatory role. Vossel et al. (2014) proposed that stronger collaboration within right‐hemisphere attention networks enhances attentional reorienting and leads to faster RTs in tasks requiring disengagement. Our findings extend this by suggesting that greater cross‐hemispheric connectivity may further facilitate the rapid reallocation of attention to new targets. This increased connectivity likely reflects greater neural efficiency, as individuals with stronger interhemispheric connections appear to disengage attention from cues with less effort.

## 5 Conclusion

Together, our findings highlight the complementary roles of occipital and parietal regions in attentional orienting. While stronger occipital‐attention network connectivity supports sustained focus on visual stimuli—potentially making disengagement more challenging—enhanced parietal‐attention network connectivity promotes attentional control, facilitating more efficient disengagement. These interactions illustrate a dynamic balance between maintaining attentional focus and enabling adaptive shifts in attention as task demands change.

## Author Contributions

**Sélim Yahia Coll**: formal analysis, investigation, data curation, writing – original draft, visualization. **Emilie Marti**: investigation, data curation, writing – review and editing. **Naz Doganci**: investigation, data curation, writing – review and editing. **Radek Ptak**: conceptualization, methodology, resources, writing – review and editing, supervision, project administration, funding acquisition.

## Ethics Statement

This study complied with the tenets of the Declaration of Helsinki and was approved by the Ethical Commission of the Canton of Geneva.

## Conflicts of Interest

The authors declare no conflicts of interest

## Peer Review

The peer review history for this article is available at https://publons.com/publon/10.1002/brb3.70868.

## Data Availability

Data will be made available on request.

## References

[brb370868-bib-0001] Becker, S. P. , A. Braimah , J. A. Dudley , L. Tamm , and J. N. Epstein . 2024. “Resting‐State Functional Connectivity in a Community Sample of Children With a Range of Cognitive Disengagement Syndrome Symptoms.” JAACAP Open 3, no. 3: 725–735. 10.1016/j.jaacop.2024.09.003. 40922795 PMC12414302

[brb370868-bib-0002] Behzadi, Y. , K. Restom , J. Liau , and T. T. Liu . 2007. “A Component Based Noise Correction Method (CompCor) for BOLD and Perfusion Based fMRI.” Neuroimage 37, no. 1: 90–101. 10.1016/j.neuroimage.2007.04.042. 17560126 PMC2214855

[brb370868-bib-0003] Benjamini, Y. , and D. Yekutieli . 2005. “False Discovery Rate–Adjusted Multiple Confidence Intervals for Selected Parameters.” Journal of the American Statistical Association 100, no. 469: 71–81. 10.1198/016214504000001907.

[brb370868-bib-0004] Bourgeois, A. , E. Marti , A. Schnider , and R. Ptak . 2022. “Task Relevance and Negative Reward Modulate the Disengagement Deficit of Patients With Spatial Neglect.” Neuropsychologia 175: 108365. 10.1016/j.neuropsychologia.2022.108365. 36058282

[brb370868-bib-0005] Carter, A. R. , M. P. McAvoy , J. S. Siegel , et al. 2017. “Differential White Matter Involvement Associated With Distinct Visuospatial Deficits After Right Hemisphere Stroke.” Cortex; A Journal Devoted to the Study of the Nervous System and Behavior 88: 81–97. 10.1016/j.cortex.2016.12.009. 28081452 PMC5462627

[brb370868-bib-0006] Cieslik, E. C. , V. I. Mueller , C. R. Eickhoff , R. Langner , and S. B. Eickhoff . 2015. “Three Key Regions for Supervisory Attentional Control : Evidence From Neuroimaging Meta‐Analyses.” Neuroscience and Biobehavioral Reviews 48: 22–34. 10.1016/j.neubiorev.2014.11.003. 25446951 PMC4272620

[brb370868-bib-0007] Coll, S. Y. , E. Marti , N. Doganci , and R. Ptak . 2024. “The Disengagement Deficit After Right‐Hemisphere Damage: Distinct Roles of Lateral Frontal and Parietal Damage.” Brain Research Bulletin 214: 111003. 10.1016/j.brainresbull.2024.111003. 38852652

[brb370868-bib-0008] Corbetta, M. , J. M. Kincade , J. M. Ollinger , M. P. McAvoy , and G. L. Shulman . 2000. “Voluntary Orienting Is Dissociated From Target Detection in Human Posterior Parietal Cortex.” Nature Neuroscience 3: 292–297. 10.1038/73009. 10700263

[brb370868-bib-0009] Corbetta, M. , G. Patel , and G. L. Shulman . 2008. “The Reorienting System of the Human Brain: From Environment to Theory of Mind.” Neuron 58: 306–324. 10.1016/j.neuron.2008.04.017. 18466742 PMC2441869

[brb370868-bib-0010] Corbetta, M. , and G. L. Shulman . 2002. “Control of Goal‐Directed and Stimulus‐Driven Attention in the Brain.” Nature Reviews Neuroscience 3, no. 3: 201–215. 10.1038/nrn755. 11994752

[brb370868-bib-0011] Fan, J. , B. D. McCandliss , J. Fossella , J. I. Flombaum , and M. I. Posner . 2005. “The Activation of Attentional Networks.” Neuroimage 26: 471–479. 10.1016/j.neuroimage.2005.02.004. 15907304

[brb370868-bib-0012] Fox, M. D. , M. Corbetta , A. Z. Snyder , J. L. Vincent , and M. E. Raichle . 2006. “Spontaneous Neuronal Activity Distinguishes Human Dorsal and Ventral Attention Systems.” Proceedings of the National Academy of Sciences 103, no. 26: 10046–10051. 10.1073/pnas.0604187103. PMC1480402 16788060

[brb370868-bib-0013] Friedrich, F. J. , R. Egly , R. D. Rafal , and D. Beck . 1998. “Spatial Attention Deficits in Humans: A Comparison of Superior Parietal and Temporal‐Parietal Junction Lesions.” Neuropsychology 12, no. 2: 193–207. 10.1037/0894-4105.12.2.193. 9556766

[brb370868-bib-0014] Golay, L. , A. Schnider , and R. Ptak . 2008. “Cortical and Subcortical Anatomy of Chronic Spatial Neglect Following Vascular Damage.” Behavioral and Brain Functions 4: 43. 10.1186/1744-9081-4-43. 18808713 PMC2557007

[brb370868-bib-0015] Han, S. W. , and R. Marois . 2014. “Functional Fractionation of the Stimulus‐Driven Attention Network.” Journal of Neuroscience 34, no. 20: 6958–6969. 10.1523/JNEUROSCI.4975-13.2014. 24828649 PMC4099520

[brb370868-bib-0016] Hopfinger, J. B. , M. H. Buonocure , and G. R. Mangun . 2000. “The Neural Mechanisms of Top‐Down Attentional Control.” Nature Neuroscience 3, no. 3: 284–291. 10.1038/72999. 10700262

[brb370868-bib-0017] Husain, M. , and C. Kennard . 1996. “Visual Neglect Associated With Frontal Lobe Infarction.” Journal of Neurology 243, no. 9: 652–657. 10.1007/BF00878662. 8892067

[brb370868-bib-0018] Indovina, I. , and E. Macaluso . 2007. “Dissociation of Stimulus Relevance and Saliency Factors During Shifts of Visuospatial Attention.” Cerebral Cortex 17: 1701–1711. 10.1093/cercor/bhl081. 17003078

[brb370868-bib-0019] Käsbauer, A.‐S. , P. Mengotti , G. R. Fink , and S. Vossel . 2020. “Resting‐State Functional Connectivity of the Right Temporoparietal Junction Relates to Belief Updating and Reorienting During Spatial Attention.” Journal of Cognitive Neuroscience 32, no. 6: 1130–1141. 10.1162/jocn_a_01543. 32027583

[brb370868-bib-0020] Kastner, S. , M. A. Pinsk , P. De Weerd , R. Desimone , and L. G. Ungerleider . 1999. “Increased Activity in Human Visual Cortex During Directed Attention in the Absence of Visual Stimulation.” Neuron 22, no. 4: 751–761. 10.1016/S0896-6273(00)80734-5. 10230795

[brb370868-bib-0021] Kincade, J. M. , R. A. Abrams , S. V. Astafiev , G. L. Shulman , and M. Corbetta . 2005. “An Event‐Related Functional Magnetic Resonance Imaging Study of Voluntary and Stimulus‐Driven Orienting of Attention.” Journal of Neuroscience 25, no. 18: 4593–4604. 10.1523/JNEUROSCI.0236-05.2005. 15872107 PMC6725019

[brb370868-bib-0022] Lissek, S. , and M. Tegenthoff . 2021. “Higher Functional Connectivity Between Prefrontal Regions and the Dorsal Attention Network Predicts Absence of Renewal.” Behavioural Brain Research 412: 113413. 10.1016/j.bbr.2021.113413. 34119509

[brb370868-bib-0023] Losier, B. J. , and R. M. Klein . 2001. “A Review of the Evidence for a Disengage Deficit Following Parietal Lobe Damage.” Neuroscience & Biobehavioral Reviews 25, no. 1: 1–13. 10.1016/S0149-7634(00)00046-4. 11166074

[brb370868-bib-0024] Machner, B. , L. Braun , J. Imholz , et al. 2022. “Resting‐State Functional Connectivity in the Dorsal Attention Network Relates to Behavioral Performance in Spatial Attention Tasks and May Show Task‐Related Adaptation.” Frontiers in Human Neuroscience 15: 757128. 10.3389/fnhum.2021.757128. 35082607 PMC8784839

[brb370868-bib-0025] Markett, S. , M. Reuter , C. Montag , et al. 2014. “Assessing the Function of the Fronto‐Parietal Attention Network: Insights From Resting‐State fMRI and the Attentional Network Test.” Human Brain Mapping 35, no. 4: 1700–1709. 10.1002/hbm.22285. 23670989 PMC6869384

[brb370868-bib-0026] Mesulam, M. M. , A. C. Nobre , Y. H. Kim , T. B. Parrish , and D. R. Gitelman . 2001. “Heterogeneity of Cingulate Contributions to Spatial Attention.” Neuroimage 13, no. 6: 1065–1072. 10.1006/nimg.2001.0768. 11352612

[brb370868-bib-0027] Morrow, L. A. , and G. Ratcliff . 1988. “The Disengagement of Covert Attention and the Neglect Syndrome.” Psychobiology 16, no. 3: 261–269. 10.3758/BF03327316.

[brb370868-bib-0028] Müller, H. J. , and J. M. Findlay . 1988. “The Effect of Visual Attention on Peripheral Discrimination Thresholds in Single and Multiple Element Displays.” Acta Psychologica 69: 129–155. 10.1016/0001-6918(88)90003-0. 3245479

[brb370868-bib-0029] Nieto‐Castanon, A. 2020. Handbook of Functional Connectivity Magnetic Resonance Imaging Methods in CONN. Hilbert Press.

[brb370868-bib-0030] Pedrazzini, E. , and R. Ptak . 2019. “Damage to the Right Temporoparietal Junction, but Not Lateral Prefrontal or Insular Cortex, Amplifies the Role of Goal‐Directed Attention.” Scientific Reports 9, no. 1: 306. 10.1038/s41598-018-36537-3. 30670788 PMC6342971

[brb370868-bib-0031] Pedrazzini, E. , and R. Ptak . 2020. “The Neuroanatomy of Spatial Awareness: A Large‐Scale Region‐of‐Interest and Voxel‐Based Anatomical Study.” Brain Imaging and Behavior 14: 615–626. 10.1007/s11682-019-00213-5. 31938986

[brb370868-bib-0032] Posner, M. I. 1980. “Orienting of Attention.” Quarterly Journal of Experimental Psychology 32, no. 1: 3–25. 10.1080/00335558008248231. 7367577

[brb370868-bib-0033] Posner, M. I. , and S. E. Petersen . 1990. “The Attention System of the Human Brain.” Annual Review of Neuroscience 13, no. 1: 25–42. 10.1146/annurev.ne.13.030190.000325 2183676

[brb370868-bib-0034] Posner, M. I. , J. A. Walker , F. J. Friedrich , and R. D. Rafal . 1984. “Effects of Parietal Injury on Covert Orienting of Attention.” Journal of Neuroscience 4, no. 7: 1863–1874. 10.1523/JNEUROSCI.04-07-01863.1984. 6737043 PMC6564871

[brb370868-bib-0050] Posner, M. I. 1984. “Neural systems and cognitive processes.” In G. Bower (Ed.), Advances in psychology 18: 241–251. North‐Holland. 10.1016/S0166-4115(08)62630-8.

[brb370868-bib-0035] Power, J. D. , A. Mitra , T. O. Laumann , A. Z. Snyder , B. L. Schlaggar , and S. E. Petersen . 2014. “Methods to Detect, Characterize, and Remove Motion Artifact in Resting State fMRI.” Neuroimage 84: 320–341. 10.1016/j.neuroimage.2013.08.048. 23994314 PMC3849338

[brb370868-bib-0036] Ptak, R. , and A. Bourgeois . 2024. “Disengagement of Attention With Spatial Neglect: A Systematic Review of Behavioral and Anatomical Findings.” Neuroscience & Biobehavioral Reviews 160: 105622. 10.1016/j.neubiorev.2024.105622. 38490498

[brb370868-bib-0037] Ptak, R. , and E. Pedrazzini . 2021. “Insular Cortex Mediates Attentional Capture by Behaviorally Relevant Stimuli After Damage to the Right Temporoparietal Junction.” Cerebral Cortex 31, no. 9: 4245–4258. 10.1093/cercor/bhab082. 33822912

[brb370868-bib-0038] Ptak, R. , and A. Schnider . 2010. “The Dorsal Attention Network Mediates Orienting Toward Behaviorally Relevant Stimuli in Spatial Neglect.” Journal of Neuroscience 30, no. 38: 12557–12565. 10.1523/JNEUROSCI.2722-10.2010. 20861361 PMC6633576

[brb370868-bib-0039] Rolls, E. T. 2019. “The Cingulate Cortex and Limbic Systems for Emotion, Action, and Memory.” Brain Structure and Function 224, no. 9: 3001–3018. 10.1007/s00429-019-01945-2. 31451898 PMC6875144

[brb370868-bib-0040] Russman Block, S. , A. P. King , R. K. Sripada , D. H. Weissman , R. Welsh , and I. Liberzon . 2017. “Behavioral and Neural Correlates of Disrupted Orienting Attention in Posttraumatic Stress Disorder.” Cognitive, Affective, & Behavioral Neuroscience 17, no. 2: 422–436. 10.3758/s13415-016-0488-2. 27966102

[brb370868-bib-0041] Serences, J. T. , S. Shomstein , A. B. Leber , X. Golay , H. E. Egeth , and S. Yantis . 2005. “Coordination of Voluntary and Stimulus‐Driven Attentional Control in Human Cortex.” Psychological Science 16, no. 2: 114–122. 10.1111/j.0956-7976.2005.00791.x. 15686577

[brb370868-bib-0042] Shomstein, S. , J. Lee , and M. Behrmann . 2010. “Top‐Down and Bottom‐Up Attentional Guidance: Investigating the Role of the Dorsal and Ventral Parietal Cortices.” Experimental Brain Research 206, no. 2: 197–208. 10.1007/s00221-010-2326-z. 20571784 PMC5728384

[brb370868-bib-0043] Siman‐Tov, T. , A. Mendelsohn , T. Schonberg , et al. 2007. “Bihemispheric Leftward Bias in a Visuospatial Attention‐Related Network.” Journal of Neuroscience 27, no. 42: 11271–11278. 10.1523/JNEUROSCI.0599-07.2007. 17942721 PMC6673032

[brb370868-bib-0044] Singh‐Curry, V. , and M. Husain . 2009. “The Functional Role of the Inferior Parietal Lobe in the Dorsal and Ventral Stream Dichotomy.” Neuropsychologia 47, no. 6: 1434–1448. 10.1016/j.neuropsychologia.2008.11.033. 19138694 PMC2697316

[brb370868-bib-0045] Small, D. M. , D. R. Gitelman , M. D. Gregory , A. C. Nobre , T. B. Parrish , and M. M. Mesulam . 2003. “The Posterior Cingulate and Medial Prefrontal Cortex Mediate the Anticipatory Allocation of Spatial Attention.” Neuroimage 18, no. 3: 633–641. 10.1016/s1053-8119(02)00012-5. 12667840

[brb370868-bib-0046] Vossel, S. , J. J. Geng , and G. R. Fink . 2014. “Dorsal and Ventral Attention Systems: Distinct Neural Circuits but Collaborative Roles.” Neuroscientist 20, no. 2: 150–159. 10.1177/1073858413494269. 23835449 PMC4107817

[brb370868-bib-0047] Watanabe, M. , K. Cheng , Y. Murayama , et al. 2011. “Attention but Not Awareness Modulates the BOLD Signal in the Human V1 During Binocular Suppression.” Science 334, no. 6057: 829–831. 10.1126/science.1203161. 22076381

[brb370868-bib-0048] Wen, X. , L. Yao , Y. Liu , and M. Ding . 2012. “Causal Interactions in Attention Networks Predict Behavioral Performance.” Journal of Neuroscience 32, no. 4: 1284–1292. 10.1523/JNEUROSCI.2817-11.2012. 22279213 PMC6796284

[brb370868-bib-0049] Whitfield‐Gabrieli, S. , and A. Nieto‐Castanon . 2012. “ *Conn*: A Functional Connectivity Toolbox for Correlated and Anticorrelated Brain Networks.” Brain Connectivity 2, no. 3: 125–141. 10.1089/brain.2012.0073. 22642651

